# Endoscopic Sphincterotomy for Cholecysto-Choledocholithiasis Complicates Subsequent Laparoscopic Cholecystectomy: A Retrospective Report From Sri Lanka

**DOI:** 10.7759/cureus.22698

**Published:** 2022-02-28

**Authors:** R G Malith Sachintha Nandasena, MA Chamila Lakmal, AA Pathirana, BD Gamage, TK Wijerathne, DD Weerasekera, Akshay Anand

**Affiliations:** 1 Department of Surgery, University of Sri Jayawardenapura Sri Lanka, Colombo, LKA; 2 Surgery, King George's Medical University, Lucknow, IND

**Keywords:** complication, risk factor, post ercp, open cholecystectomy, conversion

## Abstract

Objective: Published literature so far has supported the fact that patients who underwent endoscopic retrograde cholangio-pancreatography and sphincterotomy (ERCPS) had a difficult perioperative course after subsequent laparoscopic cholecystectomy. Through a retrospective study, this original report mentions statistics in a Southeast Asian population comparing the effect on conversion to open surgery in patients undergoing laparoscopic cholecystectomy after ERCPS in a university hospital in Sri Lanka.

Methods: The results of 205 patients who underwent laparoscopic cholecystectomy and 85 patients who were converted to open surgery between 2016 and 2018 were analyzed to find out whether ERCPS is a risk factor for conversion or subsequent perioperative morbidity.

Results: Demographics like age, gender and previous abdominal surgeries were comparable between the two groups. Cholecysto-choledocholithiasis and undergoing ERCPS for it were significant factors associated with conversion to open cholecystectomy.

Conclusion: Performing laparoscopic cholecystectomy after ERCPS for cholecysto-choledocholithiasis is a significant challenge and preferably should be often handled by a more experienced surgeon.

## Introduction

Endoscopic retrograde cholangio-pancreatography and sphincterotomy (ERCPS) followed by laparoscopic cholecystectomy is the preferred management protocol in patients with combined cholecysto-choledocholithiasis. Universal recommendations have stated that undertaking a keyhole surgery (laparoscopic cholecystectomy, LC) is prudent to prevent recurrent biliary symptoms in this subgroup of patients [1-5].

Published English literature has shown that LC post-ERCPS is more challenging than LC for primary cholelithiasis. The sequelae to open procedure after a previous ERCPS has been documented to be in the range of 8-55% compared to <5% in patients with isolated gallbladder stones [1-3,6-11]. A recent systematic review has cited a convincing link between some frequently reported risk factors and conversion to open surgery, such as male gender, higher age or BMI, ongoing acute cholecystitis and also unconventional findings like impacted stone in gallbladder neck, presence of Mirizzi’s syndrome, smoking, alcohol intake, body temperature, comorbidities like diabetes and hypertension, more than 72 hours delay between admission to surgery with acute cholecystitis and surgeons with less proficiency in performing laparoscopic procedures [12].

## Materials and methods

Study design

This study was conducted as a retrospective analysis of patients (n=290) who were planned for laparoscopic cholecystectomy and underwent the procedure, admitted to the University surgical unit, Colombo South Teaching Hospital Sri Lanka, a tertiary care referral centre, from 1st January 2016 to 31st December 2018. 

Patient selection (inclusion and exclusion criteria)

All patients who underwent cholecystectomy during the study period were included in this study. They were subsequently divided into two groups: Group I (laparoscopic cholecystectomy, LC) and Group II (laparoscopic converted to open cholecystectomy, LOC). Patients undergoing LC for an acute episode of cholecystitis were excluded from the study. If no ductal clearance could be obtained after multiple ERCPS, an open cholecystectomy with ductal exploration and stone extraction was performed, excluding these patients from this study. 

Common bile duct stones were strongly suspected if one or more of the following indications were present: jaundice and/or cholangitis, gallstone pancreatitis (acute abdominal pain and at least a fourfold increase of serum amylase activity), liver enzymes elevated to greater than twice normal levels (including bilirubin, alkaline phosphatase, c-glutamyl transferase, and transaminase), common bile duct (CBD) diameter > 8 mm, and/or stones detected by abdominal ultrasonography. ERCPS was done with a side-viewing duodenoscope. Selective cannulation of the CBD was performed. If necessary, sphincterotomy and stone removal were carried out. If no bile duct stones at ERC were found, symptoms subsided, and blood results normalized, ductal clearance was assumed. If not, a subsequent ERCPS were done.

Data collection - outcome indicators

All surgeons performing the LC have extensive experience in this surgical procedure (each having more than six years of surgical experience) and have long passed their learning curve (each surgeon performing more than 50 LC per annum). If a senior surgical resident is performing an LC, conversion is not carried out without the supervising surgeon. Retrospective statistics included patient demographics, diagnosis, the incidence of choledocholithiasis, ERCPS frequency, time to cholecystectomy and intraoperative reports. Conversion to open cholecystectomy was the primary endpoint of the study with other perioperative factors forming the secondary endpoints. 

Statistical analysis 

Statistical analysis of the data was performed using the Statistical Package for Social Sciences (SPSS) Mac OS version 23.0 (IBM Corp., Armonk, NY, USA). In addition to descriptive analysis, Chi-square and Mann Whitney U tests were applied for non-parametric data evaluation. P-value less than 0.05 was considered to be statistically significant. 

## Results

Patient characteristics

Among the patients who underwent cholecystectomy, 77 (26.6%) were male and 213 (73.4%) were female. Distributions of age, gender and previous abdominal surgeries were comparable between LC and LOC groups (Table 1).

**Table 1:** Summary of patients undergoing cholecystectomy for various reasons in University Hospital, Colombo Sri Lanka ERCP: endoscopic retrograde cholangio-pancreatography

	Laparoscopic Cholecystectomy (LC) N=205 (70.7%)	Laparoscopic c/t Open Cholecystectomy (LOC) N=85 (29.3%)	p value
Gender			0.081
Male	48 (23.4%)	29 (34.1%)	
Female	157 (76.6%)	56 (65.9%)	
Co-mobidities			
Diabetes	29 (14.1%)	22 (25.9%)	
Hypercholesterolemia	04 (1.9%)	07 (8.2%)	
Hypertension	07 (3.4%)	0	
Chronic Liver disease	0	01 (1.2%)	
None	165 (80.6%)	55 (64.7%)	
Previous abdominal Sx			0.207
No	85.4%	76.8%	
Surgeon			0.420
Consultant	43 (21%)	31 (36.5%)	
2nd yr Senior Resident	76 (37%)	48 (56.5%)	
1st yr Senior Resident	83 (40.5%)	05 (5.9%)	
Registrar	03 (1.5%)	01 (1.1%)	
Indication			-
Cholecysto-choledocholithiasis	25 (12.2%)	40 (47.1%)	
Calculous cholecystitis	72 (35.1%)	21 (24.7%)	
Biliary Colic	89 (43.4%)	12 (14.1%)	
Gallstone Pancreatitis	15 (7.3%)	02 (2.3%)	
Gall bladder Polyp	03 (1.5%)	01 (1.2%)	
Mucocele Gall baldder	01 (0.5%)	09 (10.6%)	
Reason for Conversion			-
Dense Adhesions	-	62 (72.9%)	
Bleeding	-	11 (12.9%)	
Bile leak	-	02 (2.4%)	
Mirrizi Syndrome	-	10 (11.8%)	
Surgery			0.0001*
Total Cholecystectomy	205 (100%)	44 (51.8%)	
Subtotal Cholecystectomy	0	41 (48.2%)	
Technique of Sx			0.001*
Anterograde	205 (100%)	19 (22.4%)	
Fundus First	0	66 (77.6%)	
ERCP			0.001*
Yes	25 (12.2%)	40 (47.1%)	
No	180 (87.8%)	45 (52.9%)	
ERCP freq			0.735
One	16 (64%)	27 (67.5%)	
Two	05 (20%)	08 (20%)	
Three	03 (12%)	04 (10%)	
Four	01 (4%)	0	
>=Five	0	01 (2.5%)	

Regarding co-morbidities, 51 (17.6%) patients had diabetes followed by 11 (3.8%) patients with hypercholesterolemia. However the presence of co-morbidities was not related to conversion.

Surgical characteristics

Presence of symptomatic cholelithiasis [biliary colic; n=93 (32%) and chronic calculus cholecystitis; n=101 (34.8%)] was the most common reason for offering surgery to patients followed by choledocholithiasis; n=65 (22.4%). Patients with cholecysto-choledocholithiasis underwent ERCP before undergoing cholecystectomy. Cholecysto-choledocholithiasis was a significant factor associated with conversion to open cholecystectomy (p=0.0001).

There were 85 (29.3%) conversions during this period. The main reason for switch to open operation was dense adhesions documented in 62 (~73%) patients. Intraoperative bleeding (n=11; 12.9%) and Mirrizi’s Syndrome (n=10, 11.8%) were other factors for conversion with bile leak, being responsible only in very few patients (2.5% only). 

Out of the 85 conversions, a majority (~52%) was completed as total cholecystectomy while in remaining patients only subtotal cholecystectomy could be done (p=0.0001). All patients undergoing LC were started with dissection of Calot’s triangle and ascertaining critical view of safety, the procedure was safely completed. However in patients undergoing conversion to open surgery, due to technical difficulties, nearly three-fourths (~77.6%) were approached with fundus first technique. 

In both groups, the median ERCPS attempt undertaken was one (range LC 1-4, LOC 1-5). The average time interval between ERCPS and LC was 13 weeks (range 2-52 weeks). The rate of conversion in patients with ERCPS was 61.5% (40/65) compared to only 20% (45/225) patients without previous ERCPS. Undergoing ERCPS for cholecysto-choledocholithiasis prior to cholecystectomy was significantly associated with rate of conversion to open surgery (p=0.001). Nevertheless the number of ERCP attempts had no statistically attributable risk for conversion to open cholecystectomy in the study population.

Being a teaching hospital, in addition to consultants, surgeries were also performed by senior residents and registrars under direct supervision of the consultants. Difficult cholecystectomies were mainly performed by consultants and second-year senior residents with conversion rates of 41.9% (n=31/74) and 38.7% (48/124). 

## Discussion

This is an original report from Sri Lanka comparing the complexity of conversion to open surgery following LC post-ERCPS for cholecysto-choledocholithiasis. Data indicated that patients who are subjected to LC post-ERCPS for cholecysto-choledocholithiasis are at significant risk of higher chances to proceed to open cholecystectomy and subsequently a difficult surgery during the intraoperative period. In the various studies so far, the above fact has been linked time and again [5,12,13]. It was also apparent that the number of repeated ERCPS attempts increased the risk to a higher degree although the difference was not substantial perhaps as a result of a smaller sample size [13].

Preceding reports have also mentioned a higher percentage of conversion and complications post-LC following ERCPS [6-9] and this study also is in line with them. Plausible reasons that might be attributed to this correlation are extensive inflammation in the main bile duct by stones when they pass via the cystic duct, concurrent acute biliary pancreatitis and cholangitis, premature release of inflammatory cytokines such as serum interleukin-2, interleukin-6 and tumour necrosis factor-alpha and the fact that with no Oddi’s sphincter left to protect, bacterial migration to main bile duct is common leading to fibrosis of the hepatoduodenal ligament. This notion of reflux and bacterial contamination is reinforced in a seminal work by Sugiyama and Atomi, who showed that the bile in these patients was culture-positive in the majority subset [14].

In our study, all the conversions were gallbladder-related reasons like dense adhesions, bile leak, presence of Mirrizi’s syndrome or bleeding in the hilar region. Likewise, conversion was significantly associated with difficult surgery (in the form of subtotal cholecystectomy) and the use of the fundus first technique. The operating surgeon’s preference to proceed in a much safe way may have been a bias for the higher use of the fundus first technique after conversion. This is in agreement with published literature that change to open technique is associated with higher perioperative morbidity [10,15].

However, conversion should not be seen as a complication but more as sequelae of laparoscopic cholecystectomy since it's a step to ensure patient safety. Lack of laparoscopic training in a residency or as an operating surgeon may also be a contributing factor for higher conversion rates [12]. Boddy et al. in their data analysis have stated lesser switches and difficulties if a surgeon with subspecialty training in hepato-pancreaticobiliary (HPB) surgery performed the LC [16]. In our study the centre is a teaching hospital, in addition to the consultants, senior residents and registrars under the direct supervision of the consultant also performed these surgeries. However, recently published data [17] substantiates the fact that trained minimal access surgery skilled HPB surgeons should perform difficult cholecystectomies, as the consequences of biliary vascular injuries are dreadful. It's high time that a formal laparoscopic training program should be enrolled in residency training for routine procedures like cholecystectomy in developing countries. 

A recent systematic review on risk factors for conversion of LC to open surgery endorsed a credible association between some frequently reported ones like male gender, advanced age, elevated BMI, concurrent acute cholecystitis, diabetes mellitus and hypertension and conversion to open procedure. A higher rate of symptomatic gallstones, inflammation and fibrosis in males than in women has been put forward as a plausible explanation for the gender association [12]. However in our study, we could not find any significant correlation with gender and risk of conversion though males had a slightly higher conversion rate. In our study diabetes was the most common morbidity in either group followed by hypercholesterolemia. The optimal time of LC after ERCPS is contentious. The median time interval between ERCPS and LC in our study was 13 weeks, mainly due to long waiting lists in the hospital in this part of the world. Several reports have contemplated that LC can be performed within six weeks to ease down the risk of interim biliary problems [6,11,18-20]. On the contrary Donkervoort et al. [9] reported that the interval between LC following ERCPS failed to influence the outcome of surgery. There has not been a consensus regarding the right interval for LC post-ERCPS, but it has been exhibited that LC within days or weeks of ERCPS is somewhat less challenging and can be performed without any major adverse events. Our study unit is the main tertiary care referral centre for bile duct injuries island-wide, there were no patients who had bile duct injury despite having a high conversion rate to open surgery.

## Conclusions

Laparoscopic cholecystectomy post-ERCPS for cholecysto-choledocholithiasis is a technically demanding surgery than performing LC in isolated cholelithiasis. The morbidity may increase post-conversion to open surgery however patient safety is prime. If risk factors for the conversion of a laparoscopic procedure could be pointed out explicitly, the morbidity and mortality related to an open procedure could be prevented. As of now, the two-stage procedure is widely accepted, nevertheless few centres have the facilities and talents to implement a one-stage procedure. Literature also established that improved outcomes have been a routine when laparoscopic cholecystectomy was performed by minimal access skilled HPB surgeons.
